# Effects of Class II division 1 malocclusion treatment with three types of fixed functional appliances

**DOI:** 10.1590/2177-6709.24.5.030-039.oar

**Published:** 2019

**Authors:** Deborah Brindeiro de Araújo Brito, José Fernando Castanha Henriques, Camilla Foncatti Fiedler, Guilherme Janson

**Affiliations:** 1 Universidade de São Paulo, Faculdade de Odontologia de Bauru, Departamento de Ortodontia (Bauru/SP, Brazil).

**Keywords:** Cephalometry, Orthodontic appliance design, Malocclusion, Angle Class II, Orthodontic appliances, functional

## Abstract

**Objective:**

This study aimed at comparing the dentoskeletal changes in patients with Class II division 1 malocclusion, treated with three types of fixed functional appliances.

**Methods:**

A sample comprising 95 patients with the same malocclusion, retrospectively selected, and divided into four groups, was used: G1 consisted of 25 patients (mean age 12.77 ± 1.24 years) treated with Jasper Jumper appliance; G2, with 25 patients (mean age 12.58 ± 1.65 years) treated with the Herbst appliance; G3, with 23 patients (mean age 12.37 ± 1.72 years) treated with the Mandibular Protraction Appliance (MPA); and a Control Group (CG) comprised of 22 untreated subjects (mean age 12.66 ± 1.12 years). Intergroup comparison was performed with ANOVA, followed by Tukey test.

**Results:**

The Jasper Jumper and the Herbst group showed significantly greater maxillary anterior displacement restriction. The Jasper Jumper demonstrated significantly greater increase in the mandibular plane angle, as compared to the control group. The MPA group demonstrated significantly greater palatal inclination of the maxillary incisors. Vertical development of the maxillary molars was significantly greater in the Herbst group.

**Conclusions:**

Despite some intergroup differences in the amount of dentoskeletal changes, the appliances were effective in correcting the main features of Class II malocclusions.

## RESUMO

**Objetivo:**

o objetivo do presente estudo foi comparar as alterações dentoesqueléticas em pacientes com má oclusão de Classe II, divisão 1, tratados com três tipos de aparelhos funcionais fixos.

**Métodos:**

a amostra compreendeu 95 pacientes, selecionados retrospectivamente e divididos em quatro grupos: G1, composto por 25 pacientes (idade média de 12,77 ± 1,24 anos), tratados com aparelho Jasper Jumper; G2, com 25 pacientes (idade média de 12,58 ± 1,65 anos), tratados com aparelho Herbst; G3, com 23 pacientes (idade média de 12,37 ± 1,72 anos), tratados com o Aparelho de Protração Mandibular (APM); e um Grupo Controle (GC), composto por 22 sujeitos não tratados (idade média de 12,66 ± 1,12 anos) que apresentavam a mesma má oclusão. A comparação intergrupos foi realizada com ANOVA, seguida do teste Tukey.

**Resultados:**

os grupos Jasper Jumper e Herbst apresentaram significativamente maior restrição de deslocamento anterior da maxila. O Jasper Jumper demonstrou aumento significativamente maior no ângulo do plano mandibular, em comparação ao grupo controle. O grupo APM demonstrou inclinação palatina significativamente maior dos incisivos superiores. O desenvolvimento vertical dos molares superiores foi significativamente maior no grupo Herbst.

**Conclusões:**

apesar de algumas diferenças intergrupos na quantidade de alterações dentoesqueléticas, os aparelhos foram eficazes na correção das principais características das más oclusões de Classe II.

## INTRODUCTION

Class II malocclusion is characterized by an incorrect relationship of maxillary and mandibular dental arches resulting from either skeletal or dental abnormalities, or even a combination of these conditions.^1^ ^-^ ^3^ It is considered as one of the most common orthodontic malocclusions.^4^

Several strategies are available for Class II treatment, and most orthodontists tend to choose a treatment protocol based on which part of the craniofacial skeleton is believed to be most affected by the appliance.^5^ Class II malocclusions in adults are usually treated by either orthognathic surgery or camouflage treatment, depending on the severity of the skeletal discrepancy.^6^ A common strategy in the treatment of Class II division 1 malocclusions in growing patients is a two-step approach. In the first phase of treatment, the sagittal jaw relationship is normalized, so Class II malocclusion is transformed into a Class I malocclusion. In the second phase of treatment, tooth positions are adjusted, usually with fixed appliances.^7^

Functional fixed appliances constitute a third alternative to treat Class II malocclusions without extraction or surgery.^6^ ^,^ ^8^ ^,^ ^9^ Fixed appliances with flexible intraoral force modules are used in the first phase of treatment.^10^ Fixed functional appliances offer several advantages, such as 24-hour-a-day usage; short-term treatment (approximately 8 to 10 months); esthetics is not adversely impacted; and no compliance issues.^11^

The Jasper Jumper is a fixed functional appliance considered to be an effective option for the treatment of Class II, division I malocclusion.^4^ ^,^ ^7^ It is made of a flexible intraoral power module, which is comparable to the Herbst appliance, with the advantage of having flexibility. Considered as excellent, due to great acceptance by patients, this appliance was developed to perform light and continuous forces for Class II correction, simulating the effects of the headgear and activator appliances.^12^

Despite its popularity, the Herbst appliance shows some disadvantages, including stiffness, requirement of a laboratory technique, use of special steel bands and/or crowns, and probability of dislocation or fracture. The mandibular protraction appliance (MPA) was developed as a homemade, low-cost alternative to the Herbst appliance.^11^

Therefore, the purpose of this study was to compare the dentoskeletal changes in three groups of patients with Class II division 1 malocclusion treated with the Jasper Jumper, Herbst or MPA associated with fixed appliances. These groups were compared with a control group of untreated subjects with similar malocclusions.

## MATERIAL AND METHODS

This retrospective study was approved by the Ethics in Research Committee of Bauru Dental School (FOB-USP) under protocol number 103/2005. All patient parents signed an informed consent to participate in the study.

The sample size was calculated considering an alpha error of 0.05 and a beta error of 0.2 to detect a mean difference of 1.5 mm in the overjet, with a standard deviation of 1.57.^13^ The sample size calculation indicated that 18 patients were required in each group.

The study sample comprised 95 subjects (73 treated, 22 untreated). Subject selection was based exclusively on the initial anteroposterior molar relationship, regardless of any other dentoalveolar or skeletal cephalometric characteristics. All patients met the following inclusion criteria: (1) Class II division 1 malocclusion with bilateral Class II molar relationship (minimum severity of one half Class II molar relationship); (2) no craniofacial syndromes or systemic diseases; (3) no tooth agenesis or missing permanent teeth; and (4) mandibular arch with minimal or no crowding. 

Graduate students treated all patients. All treatments were supervised by the same professor in the university clinic of the same orthodontic department at Bauru Dental School (FOB-USP).

**Figure 1:**
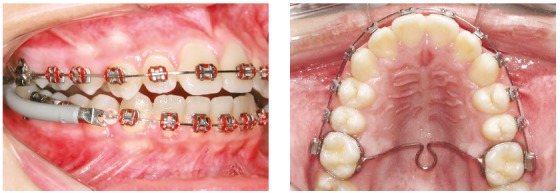
Jasper Jumper appliance installed.

The Jasper Jumper group (G1) included 25 patients (13 male, 12 female) at an initial mean age of 12.77 years, treated in a mean time of 2.15 years. Initially, fixed appliances were installed and leveling and alignment progressed until stainless steel 0.018 x 0.025-in rectangular archwires were inserted. At this stage, the Jasper Jumper appliance (American Orthodontics^®^, Sheboygan, Wl, USA) was installed to correct the Class II anteroposterior discrepancy (Fig 1). The Jasper Jumper was used until a Class I molar relationship was obtained, which took a mean of 7.32 months. Sequentially, the Jasper Jumper was removed and the patients were instructed to use Class II elastics as active retention.

**Figure 2:**
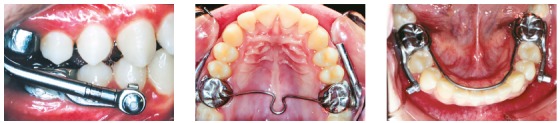
Herbst appliance installed.

The Herbst group (G2) included 25 patients (13 male, 12 female) at an initial mean age of 12.58 years, treated in a mean time of 3.11 years. The Herbst appliances (CBJ - Ormco^®^- Glendora, USA) were installed with a horizontal advancement of 6 mm, until the incisors established an edge-to-edge occlusion, and a Class I relationship was obtained (Fig 2). The Herbst appliance was used for a mean time of 18.36 months. Thereafter, fixed appliances were installed and the patients were instructed to use Class II elastics as active retention.

**Figure 3:**
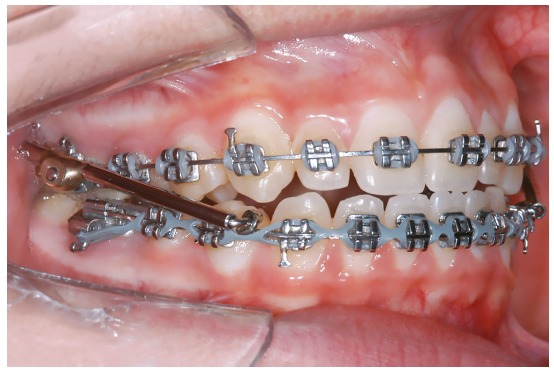
MPA installed.

The Mandibular Protraction Appliance (MPA) group (G3) consisted of 23 patients (11 male, 12 female) at an initial mean age of 12.37 years, treated in a mean time of 2.87 years. Initially, fixed appliances were installed and leveling and alignment progressed until stainless steel 0.021 x 0.025-in rectangular archwires were inserted. At this stage, the MPA appliance^14^ was installed to correct the Class II anteroposterior discrepancy (Fig 3). No transpalatal arch was used in this group. The side effects were controlled with the insertion of resistant torques in the arches. The MPA was used until a Class I molar relationship was obtained, which took a mean of 7 months. Sequentially, the MPA was removed and the patients were instructed to use Class II elastics as active retention.

All cases in the experimental groups were overcorrected.^15^

The control group (G4) was obtained from the files of the Growth Study Center at FOB-USP. This group comprised 22 subjects (12 male; 10 female) with Class II division 1 malocclusion and no orthodontic treatment, at an initial mean age of 12.66 years and a final mean age of 14.80 years, longitudinally followed-up for a mean time of 2.14 years. 

Two lateral headfilms were obtained from each patient in the following stages of orthodontic treatment: pre-treatment (T_1_) and post-treatment (T_2_), after use of the orthopedic appliance, leveling, alignment and finishing procedures.

**Figure 4:**
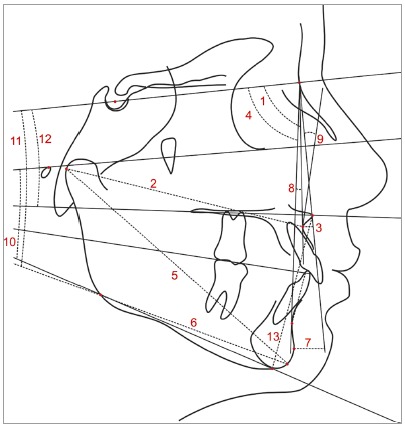
Dentoalveolar cephalometric variables: 1) SNA; 2) Co-A; 3) A-Nperp; 4) SNB; 5) Co-Gn; 6) Go-Gn; 7) Pog-Nperp; 8) ANB; 9) NAP; 10) FMA; 11) SN.GoGn; 12) SN.PP; 13) LAFH.

**Figure 5:**
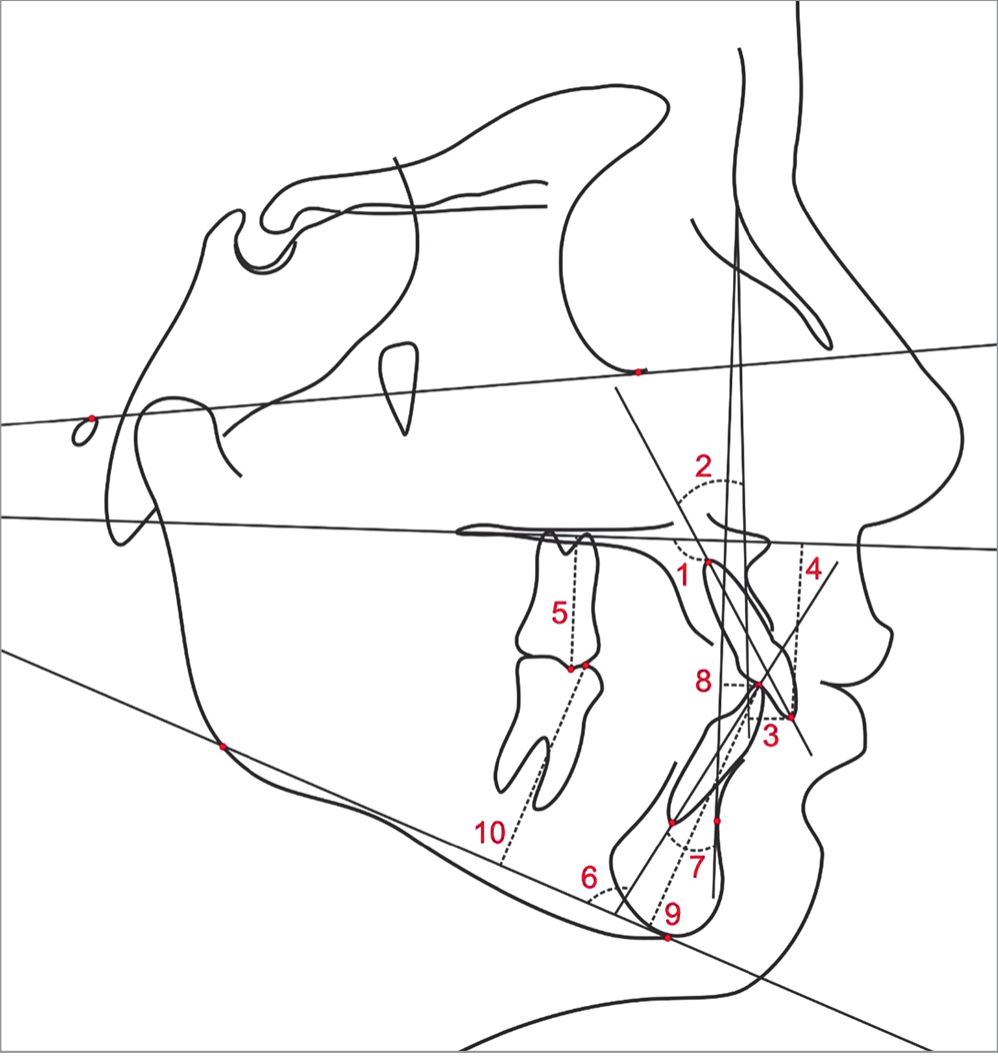
Variables related to dental relationships: 1) 1.PP; 2) 1.NA; 3) 1-NA; 4) 1-PP; 5) 6-PP; 6) IMPA; 7) 1.NB; 8) 1-NB; 9) 1-PM; 10) 6-PM.

The anatomic tracing and the location of dentoskeletal landmarks were manually carried out by a single investigator, and digitized (Numonics AccuGrid XNT, model A30TL.F, Numonics Corporation, Montgomeryville, Pa). These data were then stored in a computer and analyzed with Dentofacial Planner 7.02 (Dentofacial Planner Software Inc., Toronto, Ontario, Canada). This software also corrected the magnification factor of the radiographic images. The cephalometric variables used are defined in Table 1 and illustrated in Figures 4 and 5. 

**Table 1:** Definitions of abbreviations of the less usual cephalometric variables used.

A-N perp (mm)	Linear distance from Point A to the Nperp line (line perpendicular to the Frankfort plane passing through N)
Pog-Nperp (mm)	Linear distance between the mandibular first molar’s mesial point to the Pog-perp line (line perpendicular to the mandibular plane Go-Me passing through Pog)
Go-Me (mm)	Linear distance between the mesiovestibular cusp of the mandibular first molar perpendicular to GoMe
1.PP (degrees)	Angle formed by the maxillary incisor’s long axis and the palatal plane (PP)
1-PP (mm)	Linear distance from the maxillary central incisor edge projected perpendicularly to the PP
6-PP (mm)	Linear distance from the mesiovestibular cusp of the maxillary first molar projected perpendicularly to the PP
1.NA (degrees)	Angle formed by the maxillary incisor’s long axis and the NA line
1-NA (mm)	Linear distance between the most anterior point of the maxillary central incisor and the NA line
IMPA (degrees)	Angle formed by the mandibular incisor’s long axis and the mandibular plane (GoMe)
1.NB (degrees)	Angle formed by the mandibular incisor’s long axis and the NB line
1-NB (mm)	Linear distance between the most anterior point of the mandibular central incisor and the NB line

### Error study

A total of 30 patients were randomly selected and the radiographs were retraced, redigitized and remeasured by the same examiner after a 30-day interval. Random errors were calculated according to Dahlberg’s formula: Se^2^ = ∑d^2^/2n, where “d” is the difference between duplicate measurements and “n” is the number of double measurements. Systematic errors were estimated with paired *t* tests at significance level of *p*< 0.05. 

### Statistical analyses

Chi-square tests were used to check the comparability among the four groups regarding sex distribution and severity of the initial Class II molar relationship. Analysis of variance (ANOVA) followed by Tukey tests were used for intergroup comparisons of the initial and final ages, and initial cephalometric statuses. Intergroup treatment changes were also compared by ANOVA, followed by Tukey tests. All statistical analyses were performed with Statistica software (Statistical software for Windows, version 7.0; Statsoft, Tulsa, Okla), and the results were considered statistically significant at *p*< 0.05.

## RESULTS

The random errors were within acceptable limits and ranged from 0.23 mm (1-PP) to 0.88 mm (P-Nperp) and from 0.27^o^ (ANB) to 1.27^o^ (1.NA). Only one (SN.PP) of the 35 evaluated variables showed a statistically significant systematic error.

The initial and final ages and treatment time of the groups were compared (Table 2). To conduct direct and meaningful comparisons, due to the significant difference in treatment time between the groups, the results were annualized and all cephalometric increments of the Herbst, MPA and control groups were adjusted for the time interval of 2.15 years of the Jasper Jumper group.

**Table 2:** Mean initial and final ages and follow-up time of the study groups (ANOVA).

	GROUPS								P
Variable	Group 1 (Jasper Jumper)		Group 2 (Herbst)		Group 3 (MPA)		Group 4 (Control Group)		
	Mean	S.D.	Mean	S.D.	Mean	S.D.	Mean	S.D.	
Initial age	12.77	1.24	12.58	1.65	12.37	1.72	12.66	1.12	0.802
Final age	14.92	1.13	15.60	1.15	15.24	1.58	14.80	1.16	0.131
Follow-up	2.15^A^	0.46	3.11^B^	0.82	2.87^B^	0.77	2.14^A^	0.87	0.000*

*Statistically significant at p < 0.05.

The groups were comparable regarding sex distribution (Table 3).

**Table 3:** Comparability among the groups for sex distribution (chi-square test)

Sex	G1, Jasper Jumper (n=25)	G2, Herbst (n=25)	G3, MPA (n=23)	G4, control (n=22)	P
Male	13 (52%)	13 (52%)	11 (48%)	12 (55%)	0.975
Female	12 (48%)	12 (48%)	12 (52%)	10 (45%)	

Initial Class II anteroposterior severity was significantly smaller in the control group, but similar among the experimental groups (Table 4). 

**Table 4:** Comparability among the groups for severity of the initial anteroposterior relationship of the dental arches (Kruskal-Wallis One Way test).

Groups	Severity of Class II				P
	½ Class II	¾ Class II	Complete Class II	Total	
G1 (Jasper jumper)	4	9	12	25	0.023*
G2 (Herbst)	5	4	16	25	
G3 (APM)	6	5	12	23	
G4 (control group)	10	5	7	22	
Total	25	23	47	95	

*Statistically significant at p < 0.05.

The MPA group had a significantly greater maxillary protrusion than the other groups, while the Herbst group had the smallest maxillary and mandibular effective length and the most retruded mandible (Table 5). The maxillary incisors in the MPA group were significantly more protruded than the control group, and the maxillary molars showed significantly smaller dentoalveolar height than the Jasper Jumper group. The experimental groups had significantly greater overjet than the control group.

**Table 5:** Comparability before treatment among the groups (ANOVA and Tukey tests).

	GROUPS								
Variables	G1 (Jasper Jumper) n=25		G2 (Herbst) n=25		G3 (APM) n=23		G4 (Control Group) n=22		P
	Mean	S.D.	Mean	S.D.	Mean	S.D.	Mean	S.D.	
Maxillary component									
SNA (degrees)	82.58	3.38	82.72	3.3	82.99	4.47	81.65	3.29	0.636
A-Nperp (mm)	1.34^A^	3.63	-0.46^B^	2.64	2.91^C^	2.9	0.69^A^	2.55	0.002*
Co-A (mm)	85.87^A^	4.47	82.22^B^	3.86	84.36^AB^	4.16	87.01^A^	4.42	0.001*
Mandibular component									
SNB (degrees)	77.2	2.56	77.5	2.39	76.9	3.2	77.5	3.67	0.877
P-Nperp (mm)	-4.74^A^	5.01	-8.31^B^	3.88	-2.93^A^	4.35	-4.12^A^	4.15	0.000*
Co-Gn (mm)	106.30^A^	5.13	101.9^B^	4.75	102.90^AB^	4.78	106.80^A^	5.81	0.001*
Go-Gn (mm)	70.5^A^	3.98	65.60^B^	4.22	69.5^A^	3.71	70.20^A^	3.8	0.000*
Maxillomandibular relationship									
ANB (degrees)	5.38	2.87	5.2	2.09	6.08	2.87	4.11	1.83	0.069
NAP (degrees)	9.00^A^	7.3	8.80^A^	5.42	10.30^A^	6.77	6.90^A^	4.7	0.331
Growth pattern									
SN.GoGn (degrees)	31.1	4.01	31	4.42	31.6	5.72	30.8	4.58	0.949
FMA (degrees)	24.7	3.85	26.2	4.2	23.3	5.93	24.2	2.83	0.12
SN.PP (degrees)	7.41	2.9	5.69	3.02	7.05	4.01	8.14	3.46	0.09
LAFH (mm)	61.8	4.22	60.5	4.15	59.1	5.46	60.7	3.95	0.227
Maxillary dentoalveolar component									
1.NA (degrees)	24.50^AB^	7.3	25.20^AB^	6.98	29.30^A^	6.92	23.30^B^	6.02	0.025*
1-NA (mm)	4.64^AB^	2.57	4.80^AB^	2.32	5.95^A^	2.3	3.46^B^	1.76	0.005*
1-PP (mm)	26.5	2.61	26.3	2.34	25.4	3.03	26.6	2.53	0.389
6-PP (mm)	21.00^A^	2.12	20.00A^B^	1.43	19.50^B^	1.73	20.60^AB^	2.06	0.041*
Mandibular dentoalveolar component									
1.NB (degrees)	28.60^A^	5.83	23.60^B^	6.96	25.30^AB^	6.98	25.70^AB^	5.08	0.047*
1-NB (mm)	5.1	2.06	3.92	2.43	3.6	2.91	3.98	1.8	0.133
1-GoMe (mm)	38.6	2.84	37.1	2.59	37.2	2.81	37.2	2.4	0.141
6-GoMe (mm)	27.9	2.31	27.5	2.49	26.5	2.29	27.5	2.1	0.169
Dental relationship									
Overjet (mm)	6.24^AB^	2.21	7.09^A^	1.89	8.68^A^	2.45	4.70^B^	1.6	0.000*
Overbite (mm)	4.94	1.68	4.22	2.23	4.71	1.92	4.62	1.71	0.598

Different letters indicate statistically significant differences.

*Statistically significant at p < 0.05.

The Jasper Jumper and the Herbst groups showed significantly greater maxillary anterior displacement restriction and the Jasper Jumper also demonstrated smaller maxillary effective length increase than the control group. These two groups also presented smaller anteroposterior mandibular improvement in relation to the other groups. All the experimental groups demonstrated significant improvement of the apical base relationship in relation to the control group. The Jasper Jumper group produced increase in the vertical component. The MPA group demonstrated significantly greater palatal inclination and retrusion of the maxillary incisors in relation to the other groups. Mandibular incisor proclination was significantly greater in the Herbst and MPA groups, and its protrusion was significantly greater in the Jasper Jumper group than in the control group. Mandibular molar vertical development was significantly greater in all the experimental groups, compared to the control group. Overjet and overbite decreased significantly more in all the experimental groups than in the control group. 

## DISCUSSION

### Sample selection

The control group had less severe Class II molar relationship than the experimental group (Table 6). However, despite this limitation, a less-than-ideal control group is better than none.^7^ Comparability of the experimental groups regarding initial severity of the Class II molar relationship was a fundamental condition to compare the dentoskeletal changes, since treatment prognosis and correction of a Class II malocclusion is directly related to the initial severity of the anteroposterior discrepancy.^16^

**Table 6:** Intergroup comparison of treatment and growth changes standardized to 2.15 years (T_2_ - T_1_) (ANOVA followed by Tukey test).

	GROUPS (FINAL- INITIAL)								ANOVA
Variables	G1 (Jasper Jumper) n=25		G2 (Herbst) n=25		G3 (APM) n=23		G4 (Control Group) n=22		P
	Mean	S.D.	Mean	S.D.	Mean	S.D.	Mean	S.D.	
Maxillary component									
SNA (degrees)	-1.23^A^	2.09	-1.12^AB^	2.23	-0.07^A^	1.48	0.90^B^	2.58	0.002*
A-Nperp (mm)	-1.25^A^	2.95	-1.65^A^	2.06	0.00^AB^	1.89	1.54^B^	3.03	0.000*
Co-A (mm)	0.61^A^	2.39	1.60^AB^	2.36	2.04^AB^	1.48	2.65^B^	3.09	0.032*
Mandibular component									
SNB (degrees)	0.09	0.96	0.66	1.54	1.08	1.41	0.62	2.09	0.178
P-Nperp (mm)	-0.10^A^	4.21	-0.03^A^	2.28	1.82^B^	3.3	2.41^B^	4.69	0.043*
Co-Gn (mm)	4.04	2.81	5.86	4.54	5.41	3.27	4.48	4.42	0.328
Go-Gn (mm)	2.87	2.41	3.28	2.48	2.33	2.33	2.81	2.28	0.596
Maxillomandibular relationship									
ANB (degrees)	-1.32^A^	1.58	-1.77^A^	2.47	-1.15^A^	1.13	0.28^B^	1.21	0.000*
NAP (degrees)	-3.06^A^	3.68	-4.02^A^	5.69	-2.68^AB^	2.84	0.21^B^	2.68	0.000*
Growth pattern									
SN.GoGn (degrees)	0.57	1.49	0.07	2.09	-0.61	1.92	-0.43	1.73	0.11
FMA (degrees)	0.71^A^	2.54	0.55^AB^	1.93	-0.60^AB^	2.11	-1.08^B^	2.03	0.012*
SN.PP (degrees)	0.38	1.64	-0.23	1.79	0.09	1.64	0.37	1.66	0.548
LAFH (mm)	3.62	2.03	3.62	2.86	2.42	2.14	2.08	2.91	0.072
Maxillary dentoalveolar component									
1.NA (degrees)	-2.11^A^	8.48	-0.53^A^	7.77	-8.72^B^	9.01	-1.09^A^	2.3	0.000*
1-NA (mm)	-0.88^AB^	2.82	0.48^A^	3.01	-1.95^B^	2.44	-0.01^AB^	1.37	0.007*
1-PP (mm)	1.48	1.21	1.51	1.68	0.89	1.39	0.67	0.98	0.058
6-PP (mm)	0.96	1.23	1.99	2.37	0.97	1.69	1.7	1.31	0.092
Mandibular dentoalveolar component									
1.NB (degrees)	2.92^AB^	5.44	4.59^A^	5.05	4.85^A^	5.77	0.28^B^	4.3	0.014*
1-NB (mm)	1.56^A^	1.39	1.26^AB^	1.23	0.69^AB^	0.93	0.41^B^	1.58	0.007*
1-GoMe (mm)	0.16^A^	1.44	1.41^B^	2.09	0.18^A^	2.07	1.39^B^	2.15	0.029*
6-GoMe (mm)	2.99^A^	1.13	2.30^A^	1.69	2.63^A^	1.22	1.10^B^	1.94	0.000*
Dental relationship									
Overjet (mm)	-3.72^AB^	2.28	-2.71^A^	1.62	-4.80^B^	2.99	0.16^C^	1.25	0.000*
Overbite (mm)	-2.84^A^	1.36	-1.59^B^	1.61	-1.77^AB^	1.49	-0.25^C^	2.12	0.000*

Different letters indicate statistically significant differences;

*Statistically significant at P<0.05.

### Maxillary component

The Jasper Jumper and the Herbst appliances demonstrated greater efficiency in maxillary anterior displacement restriction. Additionally, the Jasper Jumper was also more effective in restricting maxillary effective length increase.^12^ ^,^ ^17^ ^-^ ^20^ Probably this is consequent to the more robust design of these appliances as compared with the MPA. This has also been demonstrated in other studies.^7^ ^,^ ^13^ ^,^ ^17^ ^,^ ^21^

### Mandibular component

The Jasper Jumper and Herbst groups presented smaller anteroposterior mandibular improvement, even in relation to the control group (Table 6). This is slightly different from other studies.^17^ ^,^ ^22^ ^,^ ^23^ Only the MPA showed similar anteroposterior mandibular improvement as the control group, which is similar to other studies.^24^ Although the three types of functional appliances were used to stimulate and/or redirect mandibular growth, no statistical difference was observed among the experimental and control groups concerning the mandibular length.^25^ ^,^ ^26^ Even though small differences among the experimental groups were found in these variables, they were not significantly greater than the control group. Therefore, these appliances do not seem to significantly influence mandibular growth.^27^ ^-^ ^29^

### Sagittal jaw relationship

All the experimental groups demonstrated a significantly greater improvement of the apical base relationship than the control group (Table 6). Therefore, despite some intergroup differences in the amount of maxillary growth restriction and/or in mandibular changes, the appliances are effective in correcting the Class II skeletal anteroposterior discrepancy. Other studies have also reported an improvement in the maxillomandibular relationship with the use of these appliances.^10^ ^,^ ^13^ ^,^ ^18^ ^,^ ^22^ ^,^ ^24^ ^,^ ^30^ ^-^ ^32^

### Growth pattern

Treatment with the Herbst and MPA did not cause significant vertical changes, when compared to the control group (Table 6). The Jasper Jumper group produced an increase in FMA, oppositely to the decrease shown by the control group. Even though only the Jasper Jumper change was significantly different, these results suggest that greater vertical control should be provided when using these appliances, especially in patients with some vertical growth tendency.^33^ ^-^ ^35^

### Maxillary dentoalveolar component

The MPA was the appliance that produced significantly greater palatal inclination in relation to the control group (Table 6). This could be actually consequent to the appliance effect and/or also to the non-significantly greater labial inclination and protrusion of the maxillary incisors in this group (Table 6). This result is commonly seen during the use of fixed functional appliances.^12^ ^,^ ^13^ ^,^ ^17^ ^,^ ^18^ ^,^ ^22^ ^,^ ^34^ ^,^ ^36^ However, the Herbst group produced significantly greater protrusion of the maxillary incisors than the control group. Probably there was less incisor torque control in this group, in relation to the other experimental groups.

### Mandibular dentoalveolar component

The Herbst and the MPA groups showed significantly greater proclination, and the Jasper Jumper group produced significantly greater protrusion of the mandibular incisors than the control group (Table 6). This is a common effect produced by fixed functional appliances that can be controlled by application of the necessary resistant torques.^30^ ^,^ ^32^ ^,^ ^37^ Although it is a compensatory dental positioning for Class II malocclusions, it has to be used within certain limits.^38^ There was significantly smaller extrusion of the mandibular incisors in the Jasper Jumper and MPA groups, in relation to the control group. Likewise, there was greater mandibular molar vertical development in the experimental groups than in the control group. As already mentioned, the use of functional fixed appliances tend to increase the vertical dimension and, consequently, means to control this undesirable side effects have to be planned during the orthodontic mechanics.

### Dental relationships

All the experimental groups showed significantly greater overjet and overbite reduction than the control group (Table 6), which is usually expected with these appliances.^7^ ^,^ ^13^ ^,^ ^20^ ^,^ ^38^ ^-^ ^40^ This means that although some small differences may exist in their mode of action, regarding the amount of dental skeletal changes, they will produce positive significant changes in the overjet and overbite, which are some of the important aspects to be corrected in many Class II malocclusions.^7^ ^,^ ^13^ ^,^ ^39^ ^,^ ^40^

Regarding the appliances used, new studies are needed to evaluate the long-term changes, in order to compare treatment stability with these devices.^8^

## CONCLUSIONS

The effects of the different fixed functional appliances were similar in correcting Class II malocclusion. However, the main differences observed were:

» The Jasper Jumper and the Herbst group showed significantly greater maxillary anterior displacement restriction.
» The Jasper Jumper group demonstrated significantly greater increases in the mandibular plane angle.
» The MPA group demonstrated significantly greater palatal inclination of the maxillary incisors.
